# Tuberculous Pericarditis—Own Experiences and Recent Recommendations

**DOI:** 10.3390/diagnostics12030619

**Published:** 2022-03-02

**Authors:** Małgorzata Dybowska, Katarzyna Błasińska, Juliusz Gątarek, Magdalena Klatt, Ewa Augustynowicz-Kopeć, Witold Tomkowski, Monika Szturmowicz

**Affiliations:** 1 Department of Lung Diseases, National Tuberculosis and Lung Diseases Research Institute, 01-138 Warsaw, Poland; w.tomkowski@igichp.edu.pl (W.T.); monika.szturmowicz@gmail.com (M.S.); 2 Department of Radiology, National Tuberculosis and Lung Diseases Research Institute, 01-138 Warsaw, Poland; kasiabp67@gmail.com; 3 Department of Thoracic Surgery, National Tuberculosis and Lung Diseases Research Institute, 01-138 Warsaw, Poland; j.gatarek@igichp.edu.pl; 4 Department of Microbiology, National Tuberculosis and Lung Diseases Research Institute, 01-138 Warsaw, Poland; magda.klatt@wp.pl (M.K.); e.kopec@igichp.edu.pl (E.A.-K.)

**Keywords:** tuberculous pericarditis, extrapulmonary tuberculosis, pericarditis, constrictive pericarditis

## Abstract

Tuberculous pericarditis (TBP) accounts for 1% of all forms of tuberculosis and for 1–2% of extrapulmonary tuberculosis. In endemic regions, TBP accounts for 50–90% of effusive pericarditis; in non-endemic, it only accounts for 4%. In the absence of prompt and effective treatment, TBP can lead to very serious sequelae, such as cardiac tamponade, constrictive pericarditis, and death. Early diagnosis of TBP is a cornerstone of effective treatment. The present article summarises the authors’ own experiences and highlights the current status of knowledge concerning the diagnostic and therapeutic algorithm of TBP. Special attention is drawn to new, emerging molecular methods used for confirmation of *M. tuberculosis* infection as a cause of pericarditis.

## 1. Introduction

Tuberculous pericarditis (TBP) accounts for 1% of all forms of tuberculosis and for 1–2% of extrapulmonary tuberculosis [1,2,3,4,5].

Tuberculous pericarditis, in the absence of prompt and effective treatment, can lead to very serious sequelae, such as cardiac tamponade, constrictive pericarditis, and even death [2]. According to literature, 17–40% of patients die within 6 months of diagnosis [6].

Most available data regarding TBP come from developing countries with a high tuberculosis burden and frequent coinfection with *M. tuberculosis* and HIV [7]. In these geographic regions, TBP accounts for 50–70% of effusive pericarditis in HIV-negative patients and for more than 90% in those who are HIV-positive [8]. In developed countries, TBP is rare (4% of effusive pericarditis) [9,10,11]. A majority of reported cases concern patients receiving immunosuppressive or biologic therapy [7].

Early diagnosis of TBP by microbiological methods is very difficult due to the fact that the number of mycobacteria in the pericardial fluid is low. This is the reason for the low percentage of positive acid-fast mycobacteria (AFB) smears. The confirmation of mycobacteria in the specimen by culture requires many weeks; therefore, it cannot be the basis for an early diagnosis [12]. The progress in TBP recognition is based on the introduction of molecular methods, which are based on the amplification of *M. tuberculosis*-specific genetic fragments.

It should be especially emphasised that TBP may be the only presentation of an *M. tuberculosis* infection. Thus, tuberculosis should always be taken into account in the differential diagnosis of effusive pericarditis, especially in patients who:

- Originate from regions with a large prevalence of tuberculosis;
- Are receiving immunosuppressive treatment or treatment with biological drugs;
- Are infected with HIV;
- Are diagnosed with renal insufficiency, particularly in those receiving dialysis therapy;
- Are diagnosed with diabetes mellitus;
- Are addicted to alcohol or drugs [4,13,14].

The present paper summarises the authors’ experiences concerning the diagnosis and treatment of tuberculous pericarditis and highlights the recent recommendations concerning this issue.

## 2. Clinical Vignette

A 42-year-old woman with a history of alcoholism, liver cirrhosis, arterial hypertension, and iron-deficiency anaemia was admitted to the hospital due to low-grade fever, cough, and decreased exercise tolerance.

The physical examination revealed diminished respiratory sounds at the base of both lungs. The liver was enlarged (5 cm below the costal arch); no oedema was noted in the inferior limbs.

The patient’s blood pressure was 120/90 mmHg, with a heart rate of 100 beats per min., and blood oxygen saturation measuring 94%.

Laboratory tests showed: CRP—6.7 ng/L (N < 5); leukocyte count—4.73 × 10^9^/L; erythrocyte count—4.35 × 10^12^/L, indicating mild microcytic anaemia; Hgb—9.9 g/dL; MCV—70.6 fl; and platelets count—336 × 10^9^/L.

The patient’s ECG recorded regular sinus rhythm, 110 per min., with nonspecific ST-T changes in precordial leads.

**Figure 1:**
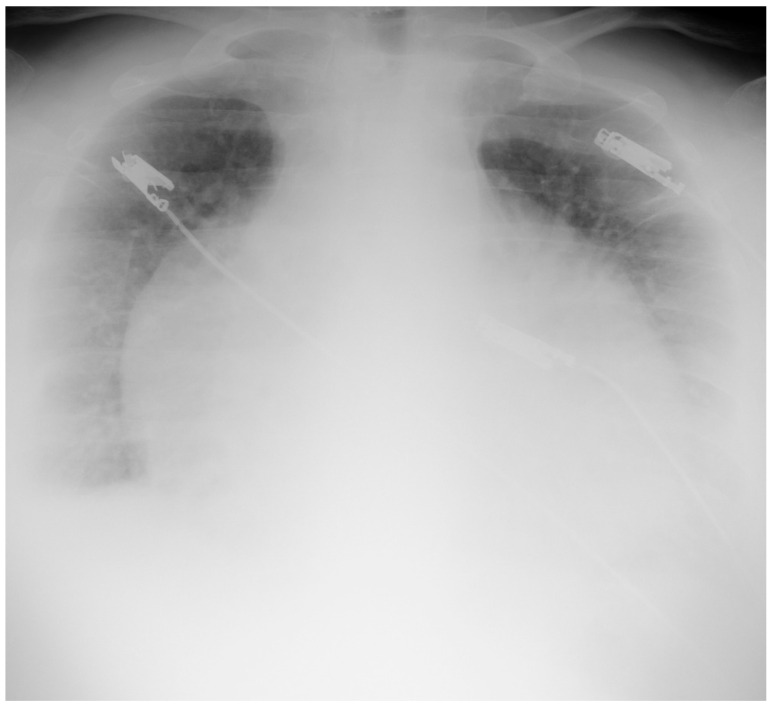
Chest radiograph in supine position shows significantly enlarged cardiac silhouette, signs of pulmonary congestion, and increased homogeneous density superimposed over the lungs due to bilateral pleural effusion.

A chest X-ray revealed a significantly enlarged heart silhouette, signs of pulmonary congestion, and a small amount of fluid in both pleural cavities. (Figure 1).

**Figure 2:**
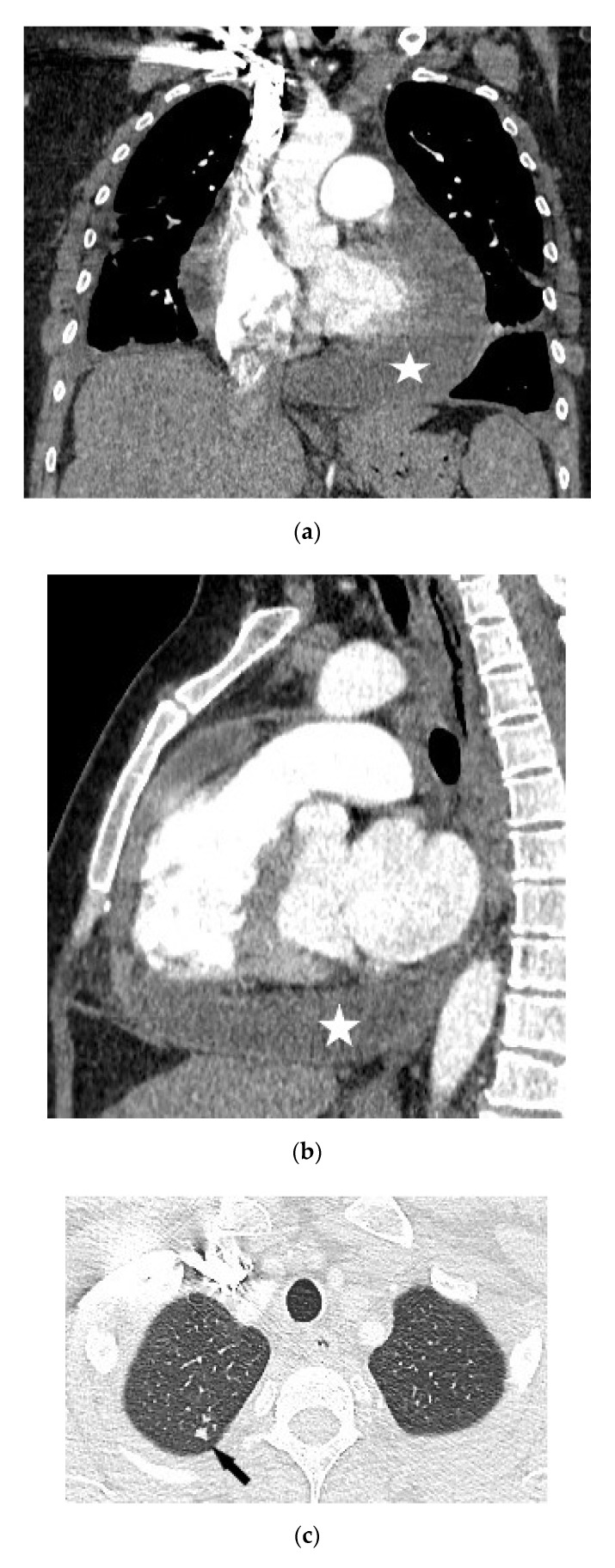

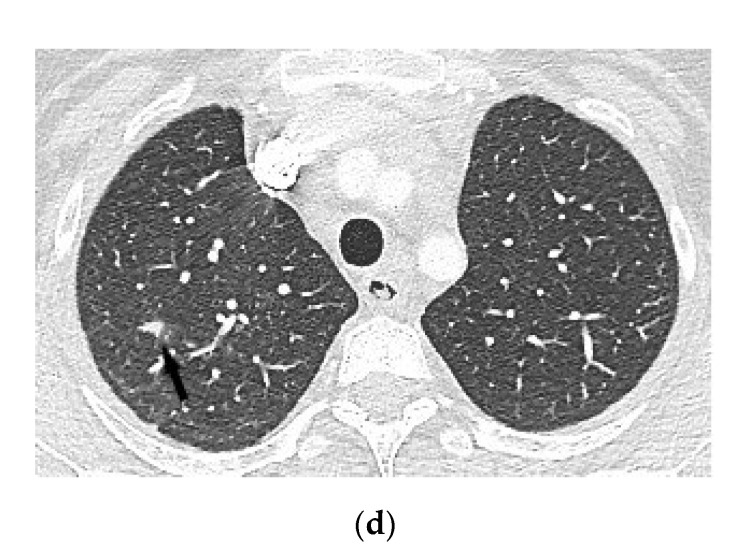
Chest CT scan with contrast enhancement: mediastinal window (**a**,**b**) and lung window (**c**,**d**) revealed large pericardial effusion up to 4 cm thick (**a**,**b**, asterisk), with no significant pericardial thickening, and SVC and IVC dilatation. A small amount of pleural fluid was also demonstrated. Additionally, several small nodules were seen in the apex of the right lung (**c**,**d**, arrows).

A chest CT revealed a large amount of fluid in the pericardium (maximal layer—43 mm), with no significant pericardial thickening and dilatation of the superior and inferior vena cava veins. Additionally, a few small nodules at the apex of the right lung and a small amount of fluid in both pleural cavities were described (Figure 2a–d).

Echocardiography showed a large amount of fluid in the pericardial sac (up to 25 mm in diastole), localised mostly behind the posterior and lateral walls of the left ventricle, with no signs of cardiac tamponade or pericardial constriction.

Bronchoscopy revealed small amounts of mucous bronchial secretions and anthracotic incrustations and scars from nodal punctures. Bacterioscopy of the bronchial washings was negative for AFB; however, the genetic material of the *Mycobacterium tuberculosis* complex was found by a GeneXpert test.

Substernal videopericardioscopy was performed. Pericardial inflammatory lesions with massive fibrin deposits were found, 750 mL of turbid fluid was evacuated, and a fragment of the pericardium was taken for histopathological examination.

A Petzer drain was left in the pericardium. During the following 6 days, about 100 mL of pericardial fluid was obtained; then, the drainage was decreased to 50 mL. The pericardial drain was removed after 8 days.

The pericardial fluid was exudate, with neutrophils predominant. An increased concentration of adenosine deaminase (ADA)—115 IU/L—was found. The cytology of the pericardial fluid was negative.

Bacterioscopy of the pericardial fluid revealed the presence of AFB. A strain of *M. tuberculosis* was cultured from the pericardial fluid, as well as from the bronchial washings, following 8 weeks of incubation.

A histopathological examination of the pericardium specimen revealed moderate, chronic inflammatory infiltrate with a single granuloma composed of epithelioid and giant cells, without signs of necrosis. A Ziehl–Neelsen staining of the pericardium specimen was negative.

Based on the presented data, a confident diagnosis of tuberculous pericarditis was made.

Antituberculous therapy was started with rifampicin, isoniazid, and ethambutol. Pyrazinamide was not used due to the patient’s liver cirrhosis. In addition, adjuvant corticosteroid therapy was administered (prednisone at an initial dose of 40 mg, then gradually tapered). The patient’s condition markedly improved. The patient is still under observation (5 years). Clinical signs of right ventricular heart failure were not observed; nevertheless, echocardiography revealed features suggestive of pericardial constriction.

## 3. Historical Group of Patients

Between 1982 and 2008, 430 patients were diagnosed with pericardial effusion at the National Tuberculosis and Lung Diseases Research Institute; tuberculous pericarditis was recognised in 11 (2.5%), and suspected in an additional 34 patients.

The criteria for TBP recognition according to ESC 2004 guidelines were as follows:

1. Positive result of a pericardial fluid culture for *M. tuberculosis*;
2. Effusive pericarditis and positive culture for *M. tuberculosis* obtained from another site;
3. Positive tuberculin skin test (TST), pericardial effusion diagnosed as lymphocytic exudate, and positive result of treatment with antituberculous drugs after the exclusion of any other cause of pericarditis [15].

The clinical, radiological, and echocardiographic data of a retrospective cohort of patients diagnosed with tuberculous pericarditis are included in Table 1 and Table 2.

The examined group consisted of seven women and five men, mean age of 58.6 ± 19.6 years.

Effusive pericarditis was diagnosed in seven patients, effusive-constrictive in three, and constrictive in one. The median of the pericardial fluid layer (echocardiography or chest CT) was 24.5 mm (3–40 mm). Echocardiographic signs of cardiac tamponade were present in two patients and features of pericardial constriction in four patients.

Invasive diagnostic and/or therapeutic procedures were applied in eight patients: pericardiocentesis in three, pericardioscopy in three, and pericardiotomy in two patients.

The median volume of drained pericardial fluid was 650 mL (120–1600 mL).

The fluid was serous in five patients and bloody in two. The median protein concentration was 5.4 g/dL (2.1–6.5 g/dL). The median lymphocytosis was 62% (7–100%). Pericardial fluid cytology was negative in all cases.

The patients were treated with antituberculous drugs, and five of them additionally received prednisone (loading dose 0.5–1 mg/kg). A decrease in pericardial fluid volume was observed in all of the patients; diminished signs of pericardial constriction were noted in three patients. Pericardiectomy was required in one patient.

## 4. Tuberculous Pericarditis—Current Diagnostic Approach

***Proposed diagnostic algorithm of tuberculous pericarditis:***

Step 1—Confirmation of pericardial effusion in imaging tests;
Step 2—Choice of further diagnostic approach;
Step 3—Confirmation of tuberculous aetiology of pericarditis.

### 4.1. Step 1: Imaging Tests

#### 4.1.1. Echocardiography

Due to its widespread availability, low cost, and mobility of diagnostic equipment, the echocardiograph remains the basic diagnostic method for pericardial diseases [7,16].

Echocardiography allows for the visualisation of fluid in the pericardial sac, determines its volume, and assesses the haemodynamic consequences of fluid accumulation [7].

In patients with tuberculous pericarditis, increased echogenicity of fluid caused by a large amount of fibrous material, and sometimes fibrous bridges, in the pericardium are visible.

Fibrin covers the pericardial layers, leading to their thickening. However, an echocardiographic examination is not optimal for measuring the thickness of the pericardium.

Echocardiography remains a key test in identifying the risk of heart tamponade, which may be visualised as:

- Collapse of the free right ventricular wall in diastole;
- Dilatation of the inferior vena cava with absence or significant limitation of its respiratory motility;
- Finding of significant respiratory variability in the tricuspid (>50%) and mitral (>25%) flow.

#### 4.1.2. Chest X-ray

Chest radiography is a routine test carried out in patients suspected of tuberculous pericarditis. It allows for the visualisation of pulmonary tuberculosis and/or pleural effusion (30–40% of patients), and occasionally, pleural calcifications (pleuritis calcarea) [1,17].

Pericardial effusion causes enlargement of the cardiac silhouette, resembling the shape of a carafe. This sign may not be present in patients with exudative–constrictive pericarditis. Chest radiography may also visualise localised calcification of pericardial layers, which are typically better seen in lateral images. The presence of calcification suggests a late stage of the disease and raises the suspicion of constrictive pericarditis. Calcifications of the pericardial sac, however, are not pathognomonic for tuberculous pericarditis and are commonly recognised in patients with idiopathic or viral pericarditis [18].

The presence of pleural fluid requires differentiation between tuberculous pleuritis and pleural transudate in the course of constrictive pericarditis.

#### 4.1.3. Chest Computed Tomography

Chest CT with contrast allows for the assessment of volume, localisation, and density of the fluid in the pericardium, as well as the thickness of the pericardium sac and the presence of possible calcifications [19].

In patients with tuberculous pericarditis, enlarged mediastinal and tracheobronchial lymph nodes (short axis > 10 mm), with a characteristic central translucency or calcification, may be present [7,20].

#### 4.1.4. Magnetic Resonance Imaging

Magnetic resonance imaging (MRI) is not widely available; therefore, its role in the diagnosis of tuberculous pericarditis is less clear. MRI does not allow for the assessment of calcification and provides worse visualisation of the lung parenchyma than CT. However, if particular conditions are met, MRI may allow for a precise assessment of the density of analysed tissues, which is important, for example, when differentiating between a small amount of pericardial fluid and thickening of the pericardial layers [19].

MRI is also helpful for recognising constrictive pericarditis, as it allows for a dynamic assessment of cardiac function in both the systolic and diastolic phases, as well as the visualisation of waves of the ventricular septum in real time. In addition, it allows for the determination of alternations of the ventricular filling during breathing (ventricular coupling assessed) [21,22].

#### 4.1.5. Fluorodeoxyglucose Positron Emission Tomography

During recent years, there has been increasing evidence of fluorodeoxyglucose positron emission tomography (18-F-FDG–PET/CT) utility in the evaluation of infectious and inflammatory disorders [23,24,25,26]. The degrees of fluorodeoxyglucose uptake in the pericardium and the mediastinal and supraclavicular lymph nodes are useful for differentiating acute tuberculous from idiopathic pericarditis [26].

### 4.2. Step 2: Choice of Further Diagnostic Approach

The diagnostic approach following the confirmation of pericarditis in diagnostic imaging depends on the volume of fluid in the pericardial sac and the clinical status of the patient.

• **In patients with signs of threatening or developed cardiac tamponade**, urgent decompression of the heart is needed [7].

Guidelines from 2015 suggest that pericardiocentesis with the evacuation of pericardial fluid should be carried out first.

• **In patients with recurrent cardiac tamponade** after pericardiocentesis, or in patients with unsuccessful pharmacotherapy and recurrent accumulation of fluid in the pericardium, it is necessary to carry out a surgical placement of a pericardial drain under general anaesthesia [7].

Surgical treatment should also be considered if there is no safe option to perform a percutaneous pericardial puncture (unfavourable location of fluid, significant obesity, significant deformation of the chest). Currently, the optimal method is substernal pericardiotomy combined with pericardioscopy, which allows for the collection of biopsy samples from the pericardial sac under visual control [27].

In the regions where tuberculosis is not endemic, it is recommended to carry out a pericardial biopsy in patients who are symptomatic for >3 weeks or if other diagnostic tests did not allow for the identification of an aetiological factor [7].

In areas where tuberculosis is endemic, carrying out a pericardial biopsy is not required prior to initiating an empirical antimycobacterial therapy [7,28].

• **In patients with a small volume of fluid in the pericardium** (<10 mm) and suspected aetiology of tuberculous pericarditis, the diagnosis should start with looking for tuberculosis at other sites.

For this purpose, bronchoscopy is performed with the collection of bronchial secretions for culture and genetic testing or bronchial biopsy, or with transbronchial lymph nodes aspiration, urine culture, and gastric lavage culture [7,17].

If enlarged cervical lymph nodes are found, a biopsy of the lymph node behind the scalene muscles should be carried out [7].

European guidelines from 2015 suggest that the following diagnostic scale should be used when there is no possibility of collecting pericardial fluid from patients from endemic regions:
Fever1 pointNight sweats1 pointLoss of body mass 2 pointsGlobulin level > 40 g/L3 pointsPeripheral leukocyte count < 10 × 10^9^/L3 points

A score of ≥6 points in the above scale is highly predictive of tuberculous pericarditis in endemic areas [7].

### 4.3. Step 3: Confirmation of Tuberculous Aetiology of Pericarditis

1. Direct Ziehl–Neelsen staining for mycobacteria [7]

Direct staining is rarely positive (0–42%) due to the paucibacillary character of the fluid [1,17,29]. There are no new methods to increase the low sensitivity of pericardial fluid smear for acid-fast mycobacteria (AFB); however, the high specificity of the bacterioscopy result justifies its continued use.

2. Cultures for *M. tuberculosis*

Pericardial fluid culture remains the most widely used diagnostic test for TBP, with sensitivity ranging from 53 to 75%; however, it takes at least 6 weeks to obtain results [1,29,30]. Cultures of sputum, bronchial aspirate, gastric aspirate, and/or urine should be considered in all patients [7].

3. Quantitative polymerase chain reaction assay (Xpert MTB/RIF) for detection of *M. tuberculosis* nucleic acids [7,31]

Molecular methods based on the amplification of *M. tuberculosis*-specific genetic fragments allow for rapid diagnosis directly from clinical specimens of patients with suspected tuberculosis (TB). At the same time, they can detect mutations in genes responsible for antimicrobial resistance. The sensitivity of genetic methods depends on the bacterial load; therefore, in smear-positive samples, the sensitivity reaches 90–100%, while in negative samples, it drops to 60–70% [32,33]. In extrapulmonary forms of TB, such as pleural, meningeal, urinary, peritoneal, and pericardial, sensitivity ranges from 50–70%. The World Health Organization has recommended the Xpert MTB/RIF molecular test for the early diagnosis of TB since 2010 [34]. This test is also applicable to extrapulmonary TB, allowing early and rapid diagnosis. However, the use of the test in the diagnosis of TBP is still being evaluated, and the diagnostic reliability of Xpert MTB/RIF for TBP compared with various reference standards is still unclear. A recent meta-analysis by Yu et al. concluded that the significance of Xpert MTB/RIF for TBP diagnosis might be different in TB-endemic and nonendemic areas. In nonendemic TB areas, the prevalence of TBP is very low, and the role of Xpert MTB/RIF still needs further investigation [35].

Because of the difficulty in establishing a diagnosis, new diagnostic methods based on molecular analyses have emerged in recent years that may be helpful in diagnosing tuberculous pericarditis in clinical practice [36,37].

Whole-genome sequencing (WGS) of bacterial genomes allows simultaneous identification of all known resistance mutations and markers to monitor transmission [38]. WGS of *M. tuberculosis* provides better resolution than other currently used methods, such as spoligotyping and mycobacterial tandem-interval repeat analysis (MIRU-VNTR) for strain genotyping [39]. Performance of WGS of *Mycobacterium tuberculosis* requires a prior culture. Currently, it is possible to sequence the whole *M. tuberculosis* genome directly from patient material [40].

4. Indirect analyses regarding tuberculosis infection: concentration of interferon-gamma, activity of adenosine deaminase, or lysozyme in pericardial fluid [7]

Adenosine deaminase (ADA) indicates the presence of stimulated monocytes and macrophages. An increased ADA concentration in body fluids, exceeding 40 IU/L, showed a sensitivity of 83–93% and a specificity of 78–97% in the recognition of tuberculous pericarditis [17,29,41]. False-positive results may occur in patients with pericarditis during the course of lymphoma, rheumatoid arthritis, or empyema. Other markers of tuberculous pericarditis with ancillary importance are interferon-gamma (>50 pg/L) and tissue lysozyme (>6.5 mcg/dL) [17,29,32,42,43,44].

5. Pericardial biopsy samples

Caseating granulomas are confirmed in 10–70% of cases with tuberculous pericarditis; however, molecular testing of pericardial biopsy samples is characterised by a greater sensitivity and specificity (80 and 100%, respectively) [1].

6. Tests assessing latent tuberculosis (LTBI)

Tuberculin skin test (TST) is of historical significance; currently, it has no significant diagnostic use, regardless of the prevalence of tuberculosis in a given region [7].

Tests based on the production of interferon-gamma via peripheral blood lymphocytes in response to stimulation from specific mycobacterial antigens (IGRAs) allow for the diagnosis of latent tuberculosis infection (LTBI), with higher specificity than TST. The role of IGRAs in the diagnosis of tuberculous pericarditis has not been specified [42].

## 5. Principles of Recognition of Tuberculous Pericarditis (ESC 2015)

Confident diagnosis:

1. Positive direct staining of pericardial fluid or pericardial biopsy specimens for mycobacteria and positive genetic test for *M. tuberculosis* of pericardial fluid;
2. Positive result of pericardial fluid or pericardial biopsy culture for *M. tuberculosis*;
3. Caseating granulomas in pericardial biopsy and positive genetic test for *M. tuberculosis*.

Probable diagnosis:

1. Active tuberculosis of another organ, confirmed with positive culture and lymphocytic pericardial effusion with increased concentration of unstimulated interferon-gamma, ADA activity, or lysozyme activity;
  and/or
2. Positive clinical response to antituberculous treatment in endemic regions.

## 6. Treatment

In every case of tuberculous pericarditis, hospitalisation of the patient is necessary, as well as initiation of antimycobacterial therapy according to generally accepted protocols. In most patients, a compound scheme of rifampicin, isoniazid, pyrazinamide, and ethambutol administered for at least 2 months, with a continuation of isoniazid and rifampicin treatment for the next 4 months, has been effective (in total, the therapy should last 6 months) [45].

Modifying the treatment is required for patients with comorbid diseases, such as hepatic or renal insufficiency, in which the treatment protocols are chosen according to specific recommendations.

An important complication of tuberculous pericarditis is constrictive pericarditis [1]. Before an effective pharmacotherapy for tuberculosis was implemented, up to 50% of individuals developed constrictive pericarditis. Treatment protocols based on rifampicin decreased the frequency of this complication to 17–40% [1,7,46]. Steroid administration as an adjuvant therapy lowers the risk of constrictive pericarditis and the necessity for hospitalisation but does not reduce mortality among patients with tuberculous pericarditis [7,28,47,48,49,50]. This benefit has been observed both in patients infected with HIV as well as HIV (−) patients [7,28,47,48,49,50]. However, the administration of steroids in HIV (+) patients increases the risk of developing secondary malignancies; therefore, adjutant steroid therapy should be implemented with caution in this group of patients [47,48,49,50].

Direct intrapericardial administration of fibrinolytic agents may be a potential method of reducing the incidence of constrictive pericarditis in patients with large tuberculous pericardial effusion. However, so far, we do not have any scientific data to support this thesis.

## 7. Prognosis

Good prognosis in patients diagnosed with tuberculous pericarditis requires early recognition and initiation of treatment. However, it is still associated with high mortality, reaching 17–40% [6]. The most important treatments are antituberculous drugs and corticosteroids. The goal of preventing pericardial fibrosis and constrictive pericarditis is of importance. Surgical pericardiectomy due to constrictive pericarditis is associated with perioperative mortality, depending on the centre, reaching 2.3–12% [51,52]. In the future, hopes are associated with intrapericardial fibrinolysis, which is currently under investigation in the Second Investigation of the Management of Pericarditis (IMPI-2) Trial (https://clinicaltrials.gov/ct2/show/ (accessed on 20 May 2018) NCT02673879). The rationale for its use is based on its theoretical ability to break up loculated fibrin strands, allowing for the complete evacuation of the pericardium and a reduction in mediators of fibrosis [2,53].

## Figures and Tables

**Table 1:** Clinical and imaging characteristics of 11 patients with tuberculous pericarditis.

No	Age (Years)	Gender	TST (mm)	Type of Pericarditis	PF Fluid Layer (mm) (CT or Echo)	Cardiac Tamponade (Echo)	Pericardial Constriction (Echo)
1	72	f	17	effusive	23		
2	52	f	22	effusive	34		
3	61	f	na	effusive-constrictive	na	+	+
4	73	m	11	constrictive	3		+
5	89	f	25	effusive	25		
6	47	m	5	effusive-constrictive	40		+
7	16	m	8	effusive	24		
8	37	m	na	effusive-constrictive	15		+
9	63	f	13	effusive	30	+	
10	58	f	0	effusive	30		
11	77	f	0	effusive	17		

Na—not available, TST—tuberculin skin test, CT—chest computed tomography, echo—echocardiography, m—male, f—female, PF—pericardial fluid, (+)—present.

**Table 2:** Results of invasive procedures performed in patients diagnosed with tuberculous pericarditis.

	Type of Procedure	Amount of Drained Fluid (mL)	Macroscopic Appearance	PF Protein Concentration (mg/dL)	Lymphocyte (%)	Positive Culture for TB (Pericardial Fluid or Other Sites)
1	pericardiotomy	600	serous	4.9	62	+
2	pericardioscopy	800	haemorrhagic	6.0	100	
3	pericardiocentesis	120	serous	2.1	7	+
4	-	-	-	-	-	
5	pericardiocentesis	700	serous	5.0	100	
6	pericardiocentesis	1600	na	na	na	
7	-	-	-	-	-	+
8	-	-	-	-	-	
9	pericardioscopy	570	serous	6.0	30	
10	pericardioscopy	900	haemorrhagic	5.4	74	
11	pericardiotomy	100	serous	6.5	20	+

PF—pericardial fluid, TB—tuberculosis, na—not available, (+)—present.

## Data Availability

Data supporting reported results can be found in source data collected in National Tuberculosis and Lung Diseases Research Institute.
